# Prediction of influenza vaccine effectiveness for the influenza season 2017/18 in the US

**DOI:** 10.12688/f1000research.13198.1

**Published:** 2017-11-29

**Authors:** Slobodan Paessler, Veljko Veljkovic

**Affiliations:** 1 Department of Pathology, Galveston National Laboratory, University of Texas Medical Branch, Galveston, TX, 77555, USA; 2 Biomed Protection, Galveston, TX, 77550, USA

**Keywords:** H3N2, influenza virus, phylogenetic analysis, seasonal influenza vaccine effectiveness

## Abstract

Vaccination against seasonal influenza viruses is the most effective way to prevent infection. A key factor in the effectiveness of the seasonal influenza vaccine is its immunological compatibility with the circulating viruses during the season. The high evolutionary rate, antigenic shift and antigenic drift of influenza viruses, represents the main obstacle for correct prediction of the vaccine effectiveness for an upcoming flu season. Conventional structural and phylogenetic approaches for assessment of vaccine effectiveness have had a limited success in prediction of vaccine efficacy in the past. Recently, a novel bioinformatics approach for assessment of effectiveness of seasonal influenza vaccine was proposed. Here, this approach was used for prediction of the vaccine effectiveness for the influenza season 2017/18 in US.

## Introduction

Influenza vaccine effectiveness (VE) can vary from year to year, and among different age and risk groups. An important factor determining VE is the similarity or “match” between the influenza viruses used in the vaccine production and the viruses circulating in the community during the flu season. Evolution of influenza viruses between the time of vaccine selection and the beginning of the flu season (week 40 for the Northern Hemisphere) can seriously hamper VE. For this reason, in most years, the flu vaccine is 50% to 70% effective ( http://www.cdc.gov/flu/professionals/vaccination/effectiveness-studies.htm; http://emergency.cdc.gov/HAN/han00374.asp), and in some years VE is <10% ^1^.

Each flu season researchers try to determine the VE adequately, but study results vary due to differences in study design, outcome(s) measured, population studied and the season in which the flu vaccine was studied. These differences make it difficult to compare the results of the several studies. However, in order to overcome these obstacles in determination of influenza VE, CDC established in 2004 the U.S. Flu Vaccine Effectiveness Network, which consists of five study sites across the United States. These study sites determine the VE by measuring the prevention of outpatient medical visits due to laboratory-confirmed influenza. The VE in the US for the period 2004–2015 determined by this Network is available.

In the 2017, flu season in Australia VE was only 10% ^2^, which resulted in very high numbers of hospitalization and deaths among vaccinated patients. Most vulnerable was the elderly population of patients (>65 years). This low VE was ascribed to variability of the H3N2 influenza viruses that were dominant in Australia that season ^2^.

Our previous phylogenetic analysis of H3N2 influenza viruses, based on a novel bioinformatics algorithm, demonstrated how variability of this virus could seriously affect VE of seasonal influenza vaccines ^3^. These results pointed out that close monitoring of evolution and spread of this virus is critical for prevention of a possible pandemic ^3^.

In this study, we compared HA1 of H3N2 viruses isolated in US and Australia in the period July/September 2017. Presented results suggest that the flu season 2017/2018 in US will be not similar to that in Australia because the VE will be likely higher. Nevertheless, this situation could be changed if the minor group of viruses, which differ from the vaccine virus, would become dominant. For this reason, close monitoring of the evolution of H3N2 viruses in US during the flu season 2017/2018 is very important.

## Methods

### Datasets

We analyzed the hemagglutinin HA1 region of 251 human H3N2 viruses collected in Australia from July to September 2017 and 113 human H3N2 viruses collected in the US in the same period (non-redundant sequences are given in Supplementary File 1 and Supplementary File 2) that were stored in publicly open database GISAID ( http://platform.gisaid.org). The vaccine virus A/Hong Kong/4801/2014 for 2017/2018 also is taken from this database.

### Informational Spectrum Method (ISM)

The ISM is a virtual spectroscopy method for the study of the informational content of the protein primary structure. This method, which is described in detail elsewhere ^4^, consists of two basic steps:

(i) Representation of amino acids in the protein primary structure with their electron-ion interaction potential (EIIP)

(ii) Conversion of the obtained numerical sequence into informational spectrum (IS) by Fourier transformation.

Frequencies in IS represent information, which is encoded by the protein primary structure, and which determines its interaction with other proteins, DNA/RNA and small molecules.

### Phylogenetic analysis

**Figure 1.**
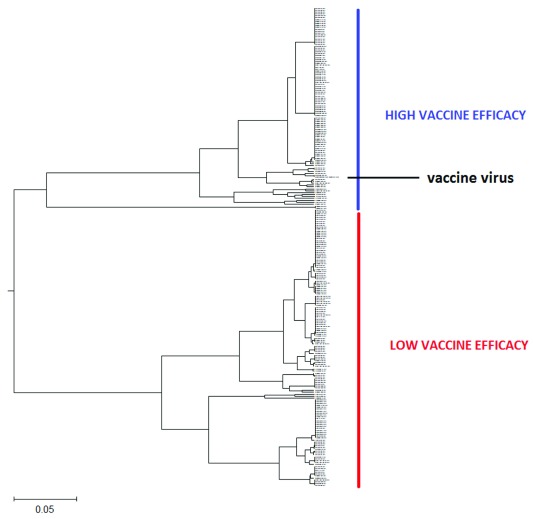
The schematic presentation of the ISM-based phylogenetic tree of HA1 from human H3N2 influenza viruses collected in Australia from July to September 2017. This tree in high resolution is given in Figure S1.

**Figure 2.**
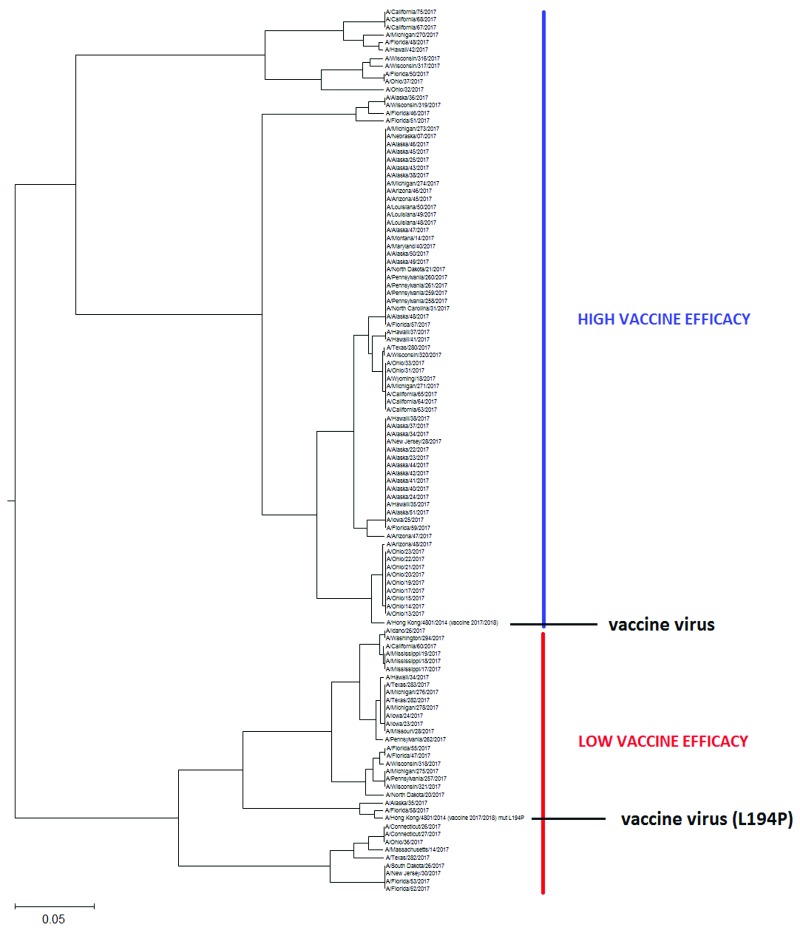
The schematic presentation of the ISM-based phylogenetic tree of the HA1 from human H3N2 influenza viruses collected in US from July to September 2017. This tree in high resolution is given in Figure S2.

The phylogenetic tree of the HA1 influenza proteins is generated with the ISM-based phylogenetic algorithm ISTREE. This algorithm was previously described in detail ^5^ (for access to ISTRE we refer the reader to http://istree.bioprotection.org). Here, we used the ISM distance measure d defined on the specific frequency F = 0.299. This frequency was extracted by ISM as the common frequency component for HA1 of H3N2 influenza viruses ^3^. The schematic presentations of calculated phylogenetic trees are given in Figure 1 and Figure 2. These trees in high resolution are given in Figure S1 and Figure S2.

## Results

Previously, it was shown that the IS frequency component F(0.299) is the informational hallmark of H3N2 viruses, which determines biological properties of HA1 of these influenza viruses ^3^. We also showed that informational complementarity represented with F(0.299) between circulating influenza viruses and the vaccine virus correlate with VE of seasonal flu vaccine ^3^.

In Figure 1 and Figure S1, the ISM-based phylogenetic tree is presented for HA1 from H3N2 viruses collected in Australia from July to September 2017 ( Supplementary File 1). We constructed this tree using the amplitude on the frequency F(0.299) as a distance matrix of the HA1 sequences. In Figure 2 and Figure S2, we present the phylogenetic tree calculated for HA1 of 113 H3N2 viruses isolated in the US between July and September 2017.

In these phylogenetic trees, we included HA1 from the vaccine virus A/Hong Kong/4801/2014.

## Discussion

Low VE of influenza vaccine in Australia prompts the US and the European health authorities to prepare for the severe influenza season 2017/2018 with suboptimal VE on the Northern hemisphere ^2^. On the other hand, there are no data or reported analysis of H3N2 viruses circulating in US in 2017 that would allow to predict the VE for the next flu season. Therefore, we performed the ISM-based phylogenetic analysis of H3N2 viruses representing precursors of seasonal flu viruses in the US. Result of this analysis served as the base for prediction of VE during the flu season 2017/2018 in the US.

As presented in Figure 1 and Figure 1S, all analyzes of Australian viruses generate two clusters and place the vaccine virus into the smaller group. This result suggests that vaccine would not be efficient against majority of Australian H3N2 viruses in the flu season 2017. This conclusion is in accord with reported low VE of influenza vaccine in Australia in 2017 flu season ^2^.

As can be seen in Figure 2 and Figure 2S, the US H3N2 viruses also are grouped into two separate clusters, however, in contrast to Australian viruses, the vaccine virus A/Hong Kong/4801/2014 belongs to the largest cluster encompassing 71% of analyzed viruses. This result suggests that VE, at least in the beginning of this flu season will not be suboptimal in the US ^2^. As for the prediction of VE in Australia, this could change during the flu season if some of the viruses from the small cluster would emerge. For this reason, continual monitoring of the evolution of H3N2 viruses is necessary.

Recently, lack in effectiveness of influenza vaccine against H3N2 viruses has been attributed to mutation L194P in HA1, which is generated during the egg-based vaccine production process ^6^. To test the effect of this mutation on the informational properties of HA1, we included this mutant of the vaccine virus in our analysis. As presented in Figure 2 and Figure 2S, this particular mutation is shifting the vaccine virus from the larger to the small cluster. This suggests that the vaccine carrying this mutation may protect only against smaller fraction of viruses during the flu season 2017/2018 and therefore result in low VE.

In summary, the presented results suggest that (i) the influenza vaccine will be effective against H3N2 viruses in the beginning of the flu season 2017/2018 in the US; (ii) it will be necessary to continue monitoring of evolution of H3N2 viruses during the flu season; and (iii) the vaccine with mutation L194P may have lower VE.

## Data availability

Sequence data of the viruses were obtained from the GISAID EpiFlu™ Database. To access the database each individual user should complete the “Registration Form For Individual Users”. This form, together with detailed instructions, are available on the website. After submission of the Registration form, the user will receive a password. There are no any other restrictions for the access to GISAID. Conditions of access to, and use of, the GISAID EpiFlu™ Database and Data are defined by Terms of Use.

## Supplementary material

**Supplementary File 1:** The names of the human H3N2 influenza viruses collected in Australia from July to September 2017, taken from GISAID ( http://platform.gisaid.org).

**Supplementary File 2:** The names of the human H3N2 influenza viruses collected in US from July to September 2017, taken from GISAID ( http://platform.gisaid.org).

**Figure S1:** The ISM-based phylogenetic tree of HA1 from human H3N2 influenza viruses collected in Australia from July to September 2017.

**Figure S2:** The ISM-based phylogenetic tree of the HA1 from human H3N2 influenza viruses collected in US from July to September 2017.
