# Supplementary figures and images for: Prediction of influenza vaccine effectiveness for the influenza season 2017/18 in the US
Source: F1000Res. 2017 Nov 29;6:2067. [Version 1] doi: 10.12688/f1000research.13198.1 (PMC5795273; doi:10.12688/f1000research.13198.1)

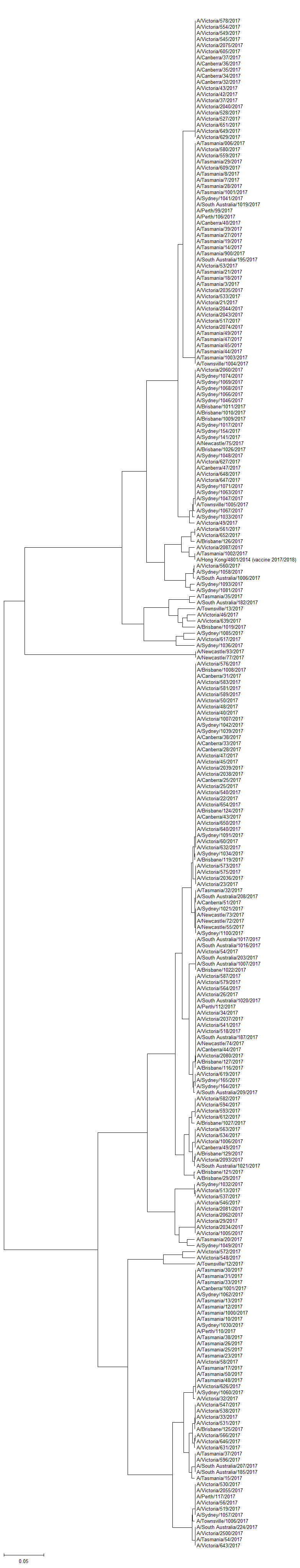

Supplement: Supplementary file 3 [file f1000research-6-14319-s0002.tgz › 67e5c3c2-f993-42da-adfa-565334c1a608.tif]

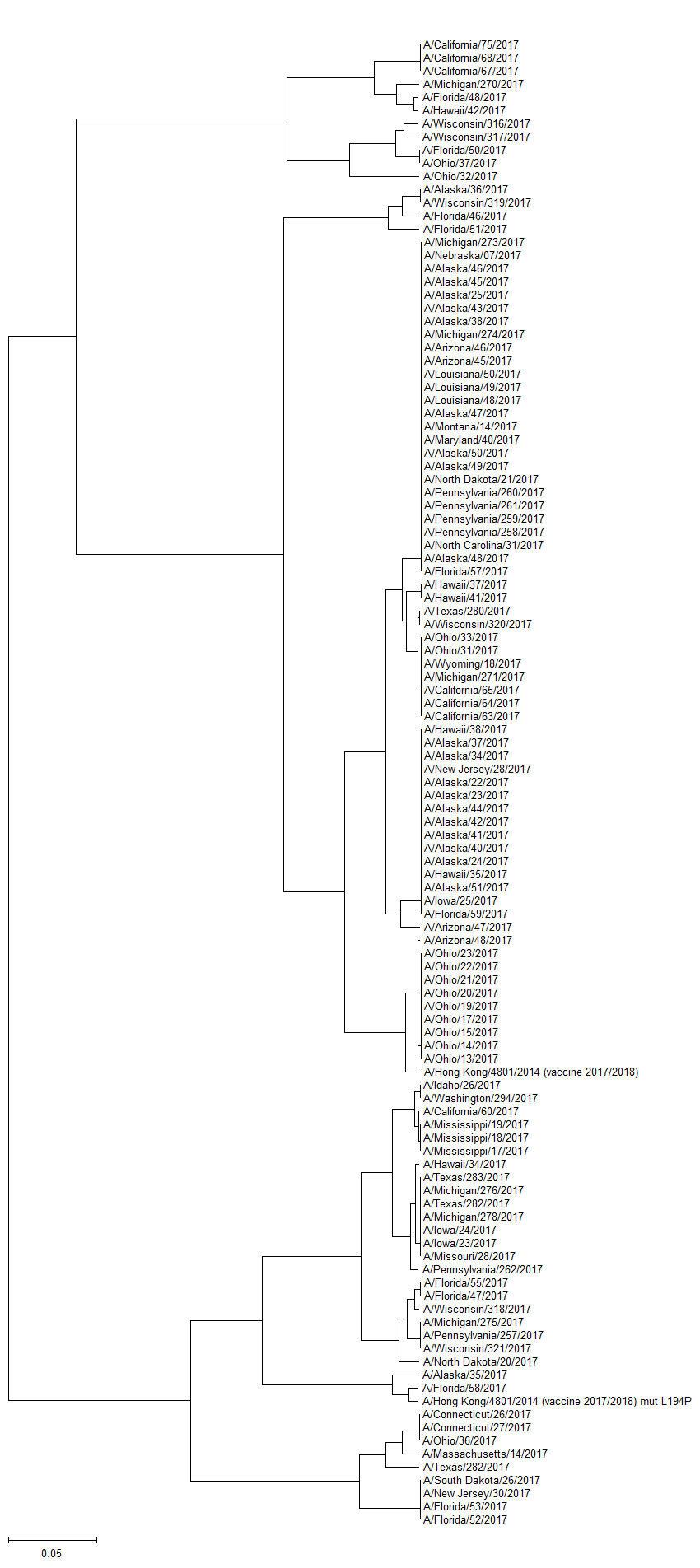

Supplement: Supplementary file 4 [file f1000research-6-14319-s0003.tgz › 9b36bbd8-8e95-4d11-9624-6e6f2a5bb9d7.tif]
