# Supplementary material for: Prediction of influenza vaccine effectiveness for the influenza season 2017/18 in the US
Source: F1000Res. 2017 Nov 29;6:2067. [Version 1] doi: 10.12688/f1000research.13198.1 (PMC5795273; doi:10.12688/f1000research.13198.1)
Supplement: Peer review history

# Peer review: Referee response for version 1

**DOI:** 10.5256/f1000research.14319.r30129

**Published:** 2018-02-01

**Authors:** Igor S. Lukashevich

**Affiliations:** 1 School of Medicine, Department of Pharmacology and Toxicology, University of Louisville, Louisville, KY, USA

This is very timing report addressing the most important public health issue, prediction of influenza vaccine efficacy. While we certainly are better prepared to flu epidemics in 2018 than in 1918, low efficiency of vaccination in recent flu season in Australia and currently ongoing flu in the US with increasing numbers of severe cases among children and elderly patients raised reasonable questions regarding prediction of correct vaccine formulations. Influenza viruses are moving targets and we have to do better job to predict and manufacture effective vaccines in timing manner. The authors applied innovative Informative Spectrum Method and analysed an extensive collection of influenza isolates from Australia and the US during July – September 2017. Based on this analysis they have predicted sub optional vaccine effectiveness for ongoing flu season in the US. The manuscript is well-written, results are solid and supported by well-developed bioinformatics tools. Discussion is appropriate. The manuscript is recommended for accelerated publication.

I have read this submission. I believe that I have an appropriate level of expertise to confirm that it is of an acceptable scientific standard.

# Peer review: Referee response for version 1

**DOI:** 10.5256/f1000research.14319.r28515

**Published:** 2017-12-18

**Authors:** Richard A. Bowen

**Affiliations:** 1 Department of Biomedical Sciences, Colorado State University, Fort Collins, CO, USA

This is a very interesting report describing a technique for computational assessment of potential vaccine efficacy of H3N2 influenza viruses. It is clear that improved techniques for predicting seasonal influenza virus vaccine efficacy are needed and this could become one of a group of evaluations that yield more precise predictions.

 I am not competent to evaluate the mathematical underpinnings of these analyses, but it would seem like some statistical analyses should be invoked; I leave it to the other reviewer(s) and editors to indicate if that is the case. The other comment I would make is that it seems as if a more historical set of evaluations would be valuable - the 2017 season is not yet over and, as the authors repeatedly indicate, things make change in terms of the viruses that come to predominate, especially in the U.S.

 Overall, I think this is a valuable contribution to forecasting for influenza virus efficacy and encourage the authors to use additional historical datasets for further evaluate the utility of the technique.

 Minor comments:

 In the 2017, flu season in Australia VE was only 10% -> In the 2017 flu season in Australia, VE was ..

 In this study, we compared HA1 of H3N2:  you haven't defined HA1 yet - might say the sequence of hemagglutinin HA1

I have read this submission. I believe that I have an appropriate level of expertise to confirm that it is of an acceptable scientific standard.
